# A Three-Component Gene Expression System and Its Application for Inducible Flavonoid Overproduction in Transgenic *Arabidopsis thaliana*

**DOI:** 10.1371/journal.pone.0017603

**Published:** 2011-03-08

**Authors:** Yue Feng, Cong-Mei Cao, Meenu Vikram, Sunghun Park, Hye Jin Kim, Jong Chan Hong, Luis Cisneros-Zevallos, Hisashi Koiwa

**Affiliations:** 1 Vegetable and Fruit Improvement Center, Department of Horticultural Science, Texas A&M University, College Station, Texas, United States of America; 2 Department of Horticulture, Forestry and Recreation Sources, Kansas State University, Manhattan, Kansas, United States of America; 3 BK21 Program, Division of Applied Life Science and Plant Molecular Biology and Biotechnology Research Center, Graduate School of Gyeongsang National University, Jinju, Korea; University of Wisconsin-Milwaukee, United States of America

## Abstract

Inducible gene expression is a powerful tool to study and engineer genes whose overexpression could be detrimental for the host organisms. However, only limited systems have been adopted in plant biotechnology. We have developed an osmotically inducible system using three components of plant origin, *RD29a* (*R*esponsive to *D*ehydration *29A*) promoter, CBF3 (*C*-repeat *B*inding *F*actor *3*) transcription factor and *cpl1-2* (*C*TD *p*hosphatase-*l*ike *1*) mutation. The osmotic stress responsible *RD29a* promoter contains the CBF3 binding sites and thus *RD29A-CBF3* feedforward cassette enhances induction of *RD29a* promoter under stress. The *cpl1-2* mutation in a host repressor *CPL1* promotes stress responsible *RD29a* promoter expression. The efficacy of this system was tested using *PAP1* (*P*roduction of *A*nthocyanin *P*igment *1*) transgene, a model transcription factor that regulates the anthocyanin pathway in Arabidopsis. While transgenic plants with only one or two of three components did not reproducibly accumulate anthocyanin pigments above the control level, transgenic *cpl1* plants containing homozygous *RD29a-PAP1* and *RD29a-CBF3* transgenes produced 30-fold higher level of total anthocyanins than control plants upon cold treatment. Growth retardation and phytochemical production of transgenic plants were minimum under normal conditions. The flavonoid profile in cold-induced transgenic plants was determined by LC/MS/MS, which resembled that of previously reported *pap1-D* plants but enriched for kaempferol derivatives. These results establish the functionality of the inducible three-component gene expression system in plant metabolic engineering. Furthermore, we show that PAP1 and environmental signals synergistically regulate the flavonoid pathway to produce a unique flavonoid blend that has not been produced by PAP1 overexpression or cold treatment alone.

## Introduction

Gain of function analysis using transgenic plants overexpressing and/or ectopically expressing a gene of interest is a commonly used strategy to understand the function of novel genes or to engineer plants for human benefits. Common obstacles in transgenic biology/biotechnology are toxicity of transgenes and instability of gene expression levels, which are often associated with constitutive overexpression of transgenes. Inducible gene expression systems are preferred in such instances, however, only a limited number of inducible gene expression systems are available for plants [1], [2]. The most popular expression systems are promoters activated by synthetic transcription factors co-expressed in the transgenic plants [1], [2], and native plant promoters activated by various environmental stimuli [3]. Typically, the former systems can strongly induce transgenes but require application of chemical inducers, whereas the expression levels achieved by the latter are lower. Enhanced induction of a plant promoter has been reported by Kasuga et al, where dehydration/cold/salt-inducible *RD29A* promoter was used to drive the expression of *CBF3* transcription factor [3]. *RD29A* promoter contains the binding sites for CBF3 protein and it was shown that a single-component, self-activation loop of *RD29A-CBF3* was sufficient to induce expressions of *CBF3* and cold-tolerance determinants specifically under low temperature [3]. This suggested a possibility to use cold-inducible plant transcription factor as a tool to potentiate the expression of transgenes under the control of cold regulated promoters.

Flavonoids are a family of compounds that are produced in both vascular and non-vascular plants. The functions of flavonoids include forming physical barriers, biochemical and visual signals to symbiotic partners and pollinators, protection from UV damage, and regulation of auxin transport during development [4], [5]. For animal consumption, flavonoids are known for health-promoting effects, displaying antioxidant activity and prevention of chronic degenerative diseases, like cancer, aging and inflammations [6], [7]. Anthocyanins are flavonoid pigments whose production is regulated by both developmental and environmental signals. Different level of anthocyanins and other flavonoids are produced under high light [8], salt stress [9], nutrient starvation [10], and cold stress [11]. The biosynthetic pathway of anthocyanin has been extensively studied in Arabidopsis, and several transcription factors including myb-type transcription factors PAP1and PAP2, and homeobox gene *Anthocyaninless2* have been identified [12], [13].

Transgenic engineering of crop plants for enhancement of anthocyanin and other flavonoids is one of the current foci of plant biotechnology to produce health-promoting functional foods. Ectopic overexpression of *PAP1* and other myb transcription factors have successfully enhanced biosynthesis of anthocyanins in various plant species [12], [14], [15], [16], [17]. Transcriptomic analysis of activation tagging mutant of *PAP1* in Arabidopsis (*pap1-D*) revealed that PAP1 strongly upregulated the expression of the anthocyanin branch of flavonoid biosynthesis pathway, while that of early phenylpropanoid pathway and flavonoid pathway was less affected [18]. These studies accomplished constitutive production of anthocyanins, however, anthocyanin and flavonoid accumulation varied substantially according to the growth condition, and the underlining mechanism for the synergy between PAP1 and environmental factors has not been fully understood. Furthermore, high level of anthocyanins/flavonoids could be inhibitory to plant growth [19], likely due to the interference of auxin transport by flavonoids [20]. In order to achieve high-level of anthocyanin production without causing growth defects, it is desirable to employ inducible production of phytochemicals that separates growth phase and production phase, and the latter could be initiated by physical and/or chemical stimuli.

Here we report a three-component gene expression system and its application to cold-inducible anthocyanin production. A gene of interest (*PAP1*) was cloned downstream of a cold-inducible *RD29A* promoter, and Arabidopsis plants were co-transformed with *RD29A-PAP1* and a feedforward effecter gene of the cold signal (*RD29A-CBF3*). We determined that a mutation in host repressor *CPL1* [21], [22] is an essential third component for the success of this expression system. Cold induction activated expressions of *PAP1* and anthocyanin biosynthetic genes, which were accompanied with overproduction of anthocyanins. The flavonoid phytochemical profiles of transgenic plants showed synergism of native and PAP1-induced flavonoid productions. Our results establish that a three-component system using a native plant promoter is sufficient to drive high expression of transgenes upon induction. We believe the system and its variations will be valuable tools to integrate plant environmental responses to a broad range of processes, such as metabolic and physiological engineering, and heterologous protein expression strategies.

## Results

### Designing osmotic-stress-inducible transcription factor cassettes

**Figure 1:**
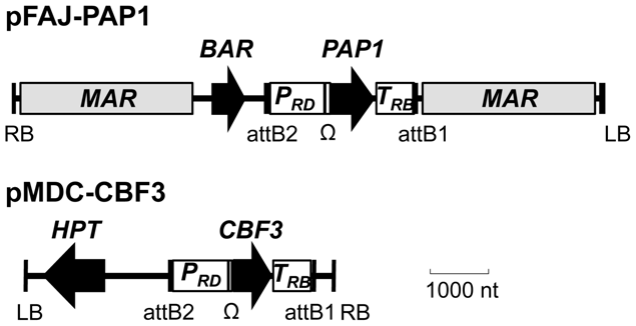
Schematic illustrations of transgene expression cassettes in pFAJ-PAP1 and pMDC-CBF3. P*_RD_*; Arabidopsis RD29a promoter, Ω; tobacco mosaic virus omega sequence, BAR; BASTA resistance gene, HPT; hygromycin phosphotransferase, MAR; chicken matrix attachment region, attB; gateway recombination sites, LB; T-DNA left border, RB; T-DNA right border, T*_RB_*; soybean Ribulose-1,5-bisphosphate carboxylase oxygenase terminator.

In order to develop and test inducible gene expression systems, we chose the phenylpropanoid pathway as a model target. The phenylpropanoid pathway in Arabidopsis is regulated by the PAP1 transcription factor and overexpression of PAP1 produces plants with easily scorable purple pigments. Resulting phytochemicals have been implicated for human health benefits. We also chose cold treatment as a trigger, since it allowed extended period of treatments compared to other signals such as heat, light, and chemical inducers. A cold-regulated *PAP1* overexpression cassette was prepared by placing the *PAP1* cDNA downstream of the *RD29A* promoter and tobacco mosaic virus *Omega* sequence (Figure 1). In order to enhance the efficacy of induction, an *RD29A-CBF3* effecter gene cassette was prepared. Since CBF3 binds to and promotes expression of the *RD29A* promoter, *RD29A-CBF3* functions as a cold-induced self-amplicon, which will feedforward the expression of the *RD29A* promoter. Furthermore, the effecter gene has a protective function during the cold treatment [3].

### Co-expression of *RD29A-PAP1* and *RD29A-CBF3* was not sufficient to induce anthocyanin accumulation by cold treatment

**Figure 2:**
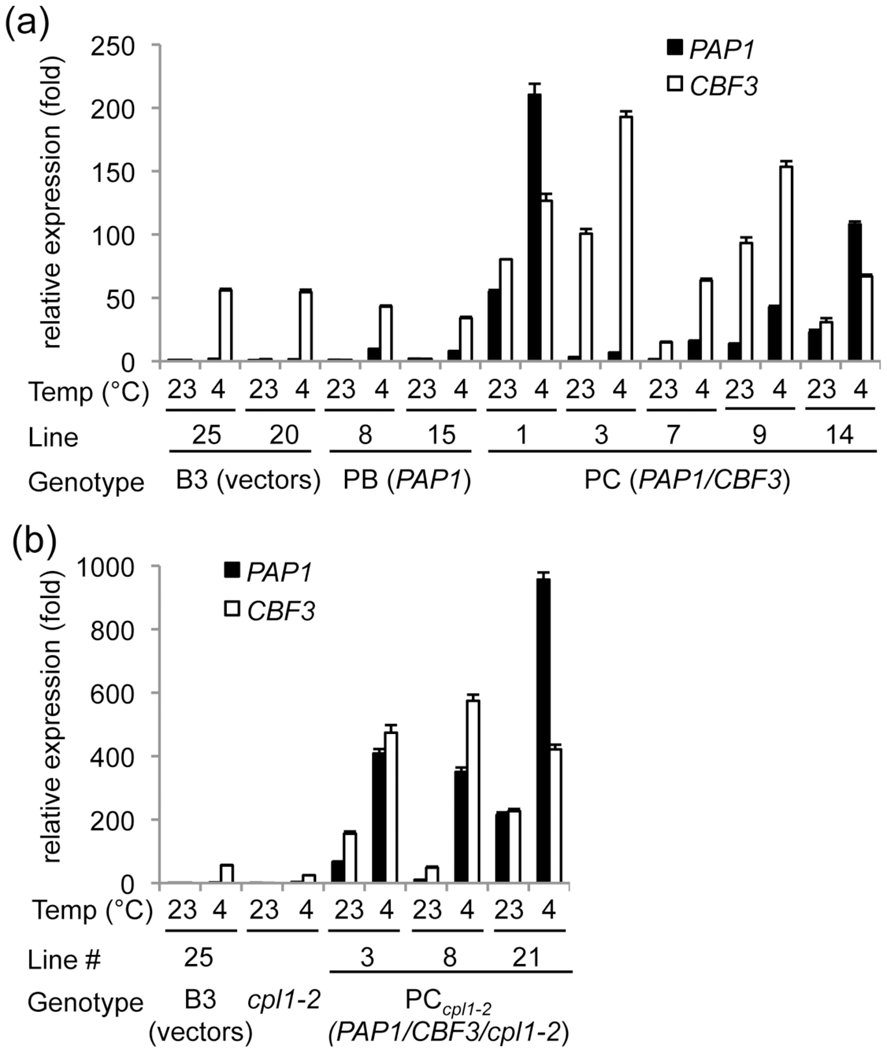
RT-qPCR analyses of *PAP1* and *CBF3* gene expressions in transgenic plants. (a) wild type background. (b) *cpl1-2* background. Total RNAs were extracted from 7-day-old seedling grown at 23°C and from seedlings treated with low temperature (4°C) for additional 4 days. Expression folds of each gene were shown relative to the levels of vector control line grown at 23°C. Bars indicate standard errors from duplicates.

The *PAP1* and/or *CBF3* expression cassettes as well as vector control constructs were introduced into wild type Arabidopsis plants. These lines were designated as B3 (vector control), PB (*RD29A-PAP1* only), PC (*RD29A-PAP1* and *RD29A-CBF3*). Plants containing each expression cassette as a single copy were selected based on hygromycin (for pMDC-CBF3) and Liberty resistance (for pFAJ-PAP1), and homozygous T_3_ lines were identified. The homozygous lines were screened for the expression of transgene and anthocyanin contents before and after cold treatment (Figure 2a, Table 1). Compared to the untreated vector control lines, cold-treated PB transformants with *RD29A-PAP1* expressed 7-10 fold higher level of *PAP1*. Pyramiding *RD29A-CBF3* on top of *RD29A-PAP1* (PC lines) enhanced the *PAP1* expression level up to 200-fold over unstressed vector control plants, indicating the *RD29A-CBF3* effecter did indeed feedforward the *RD29A* promoter activity. Surprisingly, PB, PC, and B3 lines showed a similar level of transcripts encoding phenylpropanoid pathway enzymes, such as *PAL1* (phenylalanine ammonia lyase 1) and *CHS* (chalcone synthase) (data not shown), and total anthocyanin contents (cyanidin 3-glucoside equivalent) of PB/PC lines were not substantially higher than those of vector control lines even after cold treatment for 3 weeks (Table 1).

**Table 1:** Anthocyanin levels in representative transgenic lines growing at 23°C or 4°C.

Lines	Anthocyanin (±std)	
	(µg cyanidin-3-glucoside/g tissue)	
	23°C	4°C
B3-20	31 (10)	44 (2)
B3-25	21 (6)	51 (26)
PB-8	149 (4)	31 (7)
PB-15	71 (8)	39 (2)
PC-1	34 (3)	41 (19)
PC-3	13 (1)	70 (5)
PC-7	57 (22)	54 (26)
PC-9	95 (1)	37 (21)
PC-14	56 (14)	51 (11)
PC*_cpl1_*-3	330 (3)	468 (13)
PC*_cpl1_*-8	455(0)	1371 (36)
PC*_cpl1_*-21	523 (0.7)	929 (22)

Values are mean of duplicates.

### Three-component system with *cpl2-1* background induced anthocyanin production under low temperature

**Figure 3:**
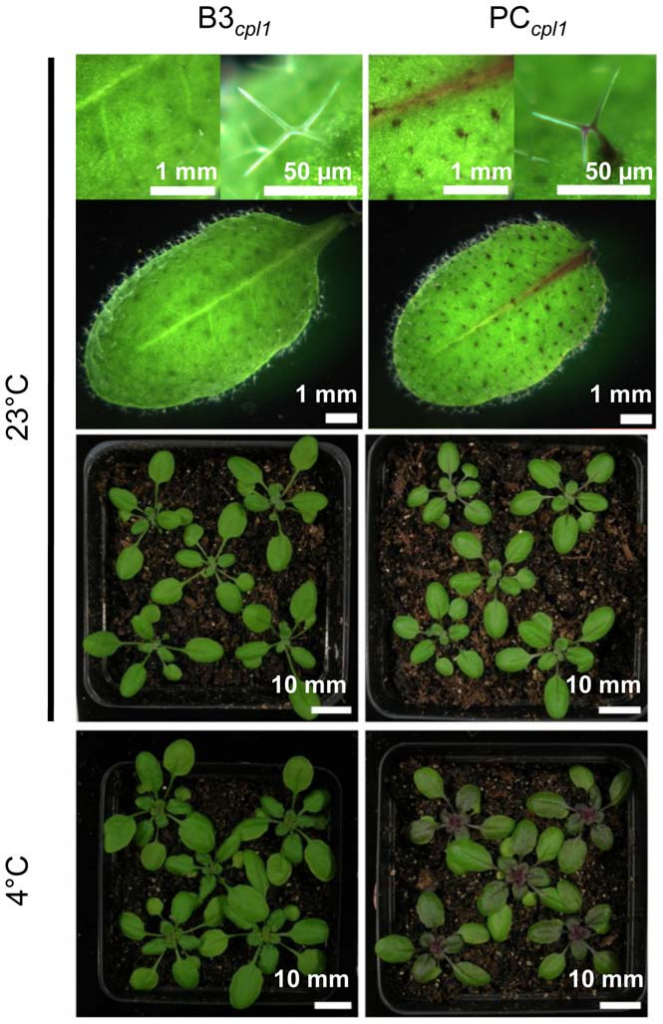
Photographs of leaves of homozygous transgenic B3*_cpl1_* and in PC*_cpl1_* plants under normal growth condition for 3 weeks (23°C) and after cold treatment (4°C) for additional 3 weeks.

We hypothesized that the lack of anthocyanin accumulation in PB and PC lines was due to insufficient level of *PAP1* expression even after cold-induction. To increase the efficacy of cold induction, we incorporated the third component, *cpl1-2* mutation. Arabidopsis host plants with *cpl1-2* mutation, which could induce *RD29A* promoter up to 10 fold higher than wild type [21], was used as a recipient of the *RD29A-PAP1* and *RD29A-CBF3* transgenes. These lines and vector control lines were designated as PC*_cpl1_* and B3*_cpl1_*, respectively, and homozygous plants were selected. *cpl1* lines containing *RD29A-PAP1* without *RD29A-CBF3* (PB*_cpl1_* lines) were also prepared, but were not characterized in detail because of the lack of visible anthocyanin production (data not shown). During the selection, we noted a frequent occurrence of PC*_cpl1_* lines with spotty pigmentations on the leaf surface. Microscopic observations showed that these spots were trichomes accumulating anthocyanin pigments (Figure 3). Partial coloration was observed in leaf veins as well. Some individuals showed high level of pigments in entire plant bodies and grew very slowly, which were not included in further analyses. RT-qPCR analyses indicated that 4 days cold treatment induced *PAP1* expression up to 950-fold in PC*_cpl1_* line over the vector control lines (Figure 2b). Total anthocyanin analyses showed that PC*_cpl1_* plants accumulated up to 30-fold more anthocyanin than vector control plants did. The levels of anthocyanin produced in PC*_cpl1_* lines were comparable to the level produced in constitutive overexpression of *PAP1* lines controlled by the cauliflower mosaic virus 35S promoter [8]. These results indicate that the three-component system is necessary to induce anthocyanin biosynthetic pathway above the threshold level. Since PC_cpl1_ line 21 consistently induced *PAP1* and anthocyanin to high level, this line was used for further analysis.

### Gene expression profile of three-component transgenic plants during cold activation

**Figure 4:**
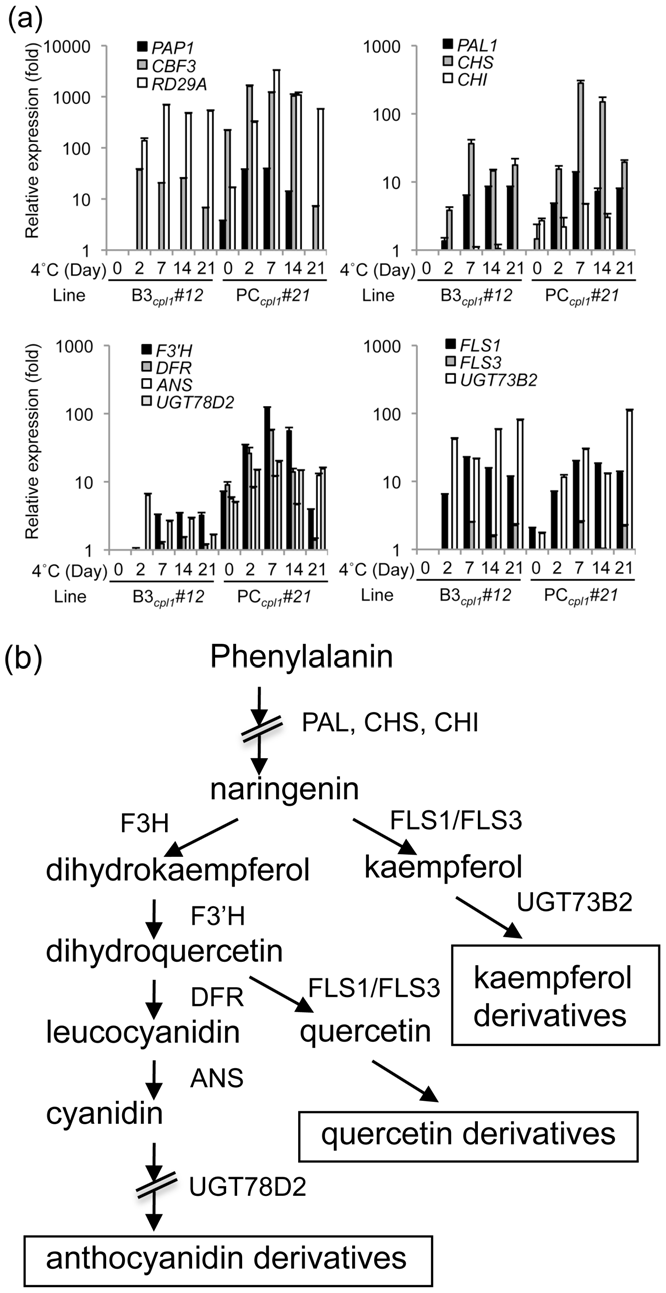
Time-course expressions of transgene and flavonoid biosynthetic pathway genes in B3*_cpl1_* and in PC*_cpl1_* lines during cold treatment. (a) Total RNAs were extracted from leaves of 3-week-old plants grown at room temperature (23°C) and plants treated with cold (4°C) for additional 2 days, 1 week, 2 weeks and 3 weeks. Expression levels of each gene were shown relative to the levels of B3*_cpl1_* grown at 23°C. Bars indicate standard errors. Experiments were conducted two times with similar results. Results from one experiment were shown. *PAL1*; phenylalanine ammonia lyase 1, *CHS*; chalcone synthase, *CHI*; chalcone isomerase, *F3′H*; flavonoid 3′-hydroxylase, *DFR*; dihydroflavonol reductase, *ANS*; anthocyanidin synthase, *FLS1*; flavonol synthase 1, *FLS3*; flavonol synthase 3, *UGT73B2*; UDP-glucosyltransferase 73B2 (flavonol 3-O-glucosyltransferase activity), *UGT78D2*; UDP-glucosyltransferase 78D2 (anthocyanidin 3-*O*-glucosyltransferase). (b) Structure of phenylpropanoid pathway. Transcript levels of the enzymes in the marked steps were analyzed in (a).

In order to understand the efficacy of the cold-inducible three-component system, a time course of gene expression was determined for transgenes and genes encoding the flavonoid biosynthesis pathway during a long-term cold induction (Figure 4). Three-week old PC*_cpl1_* and B3*_cpl1_* plants were exposed to 4°C for up to additional 3 weeks. Cold treatments longer than 3 weeks induced senescence of plants and therefore were not included in the analysis. In PC*_cpl1_*, expression of *CBF3* and *PAP1* reached their highest levels (1,600 fold and 37 fold, respectively) after 2 days and slowly declined after 1 week. After 3 weeks of cold treatment, the *PAP1* level was similar to that of vector control plants. Expression of *PAL1* (phenylalanine ammonia lyase 1) was induced both in cold-treated PC*_cpl1_* and B3*_cpl1_* plants, albeit PC*_cpl1_* plants showed slightly faster response and higher expression level. Genes that lead to anthocyanin biosynthesis, such as *CHS* (chalcone synthase), *CHI* (chalcone isomerase), *F3′H* (flavonoid 3′-hydroxylase), *DFR* (dihydroflavonol reductase), and *ANS* (anthocyanidin synthase) were all expressed at higher levels in cold-treated PC*_cpl1_* plants. In contrast, cold treatment induced expression of *FLS* (flavonol synthase) and *UGT73B2* (flavonol glucosyltransferases) both in PC*_cpl1_* and B3*_cpl1_* plants to the similar levels. These results indicated that the three-component system effectively activated the anthocyanin biosynthesis pathway, whereas cold treatment itself induced flavonol biosynthesis pathway genes independent of the three-component system. In addition, the induction of the anthocyanin biosynthesis pathway persisted until plants started to senesce after three weeks of cold treatment.

### Identification and quantification of flavonoid compounds by LC-MS

**Figure 5:**
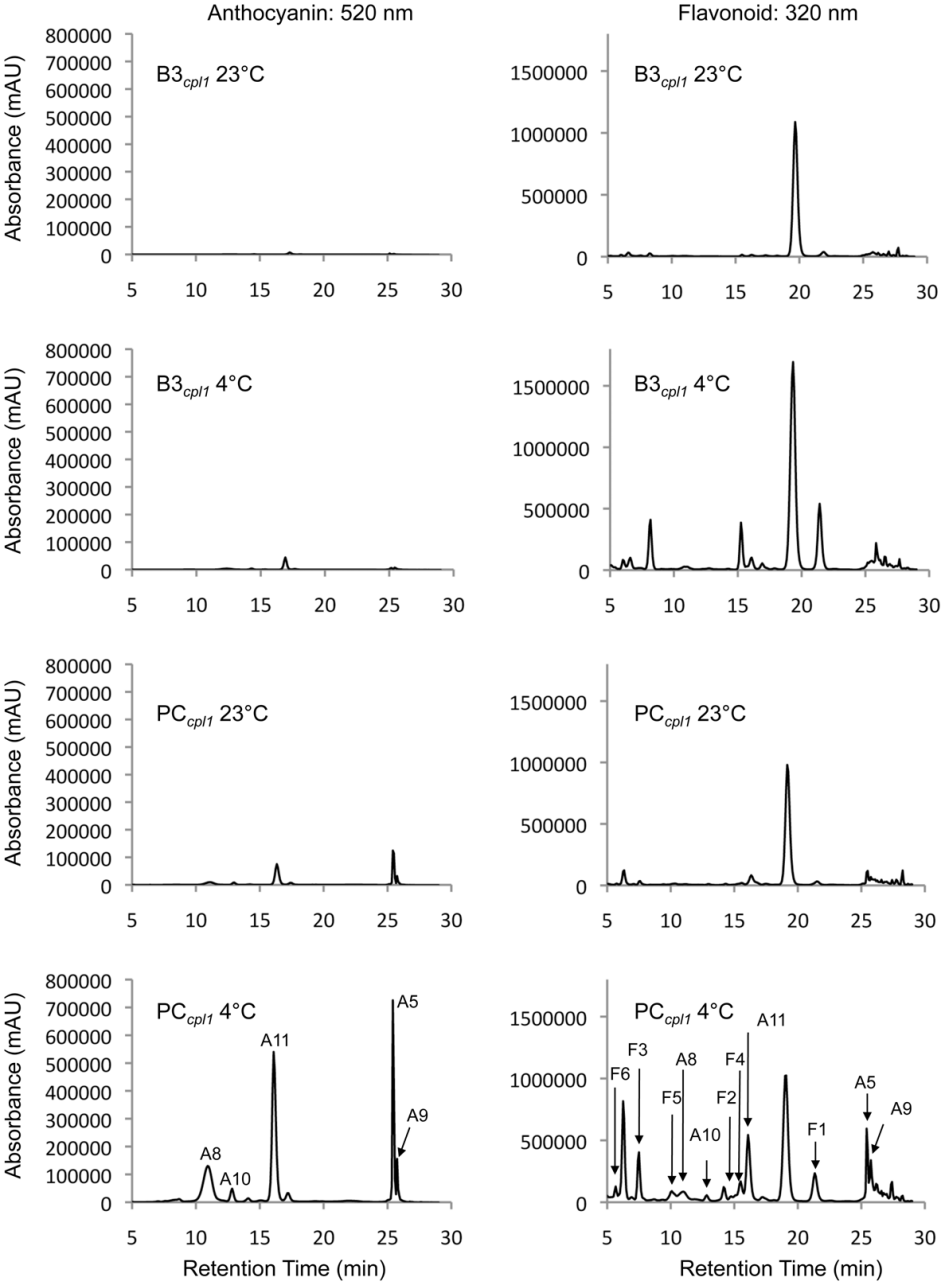
HPLC/PDA chromatograms showing the flavonoid profiles of PC*_cpl1_* and B3*_cpl1_* plants under normal growth condition (23°C) or after additional 3 weeks of cold treatment (4°C). “A” stands for cyanidin derivatives, and “F” stands for flavonoids, which were identified by LC-MS. The peaks were labeled according to Tohge et al (2005).

In PC*_cpl1_* plants, cold induction of anthocyanin biosynthesis pathway genes were accompanied with accumulation of anthocyanin pigments throughout the aerial part of plant bodies, indicating that the cold-induction system indeed increased biosynthetic capacity of anthocyanins in transgenic plants (Figure 3, bottom). In order to determine whether anthocyanin phytochemicals produced via three-component system is similar to those produced by constitutive overexpression of *PAP1*, profiles of anthocyanins and other flavonoids produced in transgenic plants were analyzed (Figure 5, Table 2). Phytochemicals were extracted from 3 weeks old PC*_cpl1_* and B3*_cpl1_* plants grown at room temperature (23°C) and plants after additional three weeks of cold treatment (4°C). Putative flavonoid compounds were identified by comparing their retention time and UV-visible absorption spectra in LC, and their molecular charge ratios and fragmentation patterns in MS/MS analyses, to the reported profiles [18]. Authentic standards were used to determine the amount of each compound using HPLC chromatogram. Five major anthocyanins (cyanidin derivatives) and six additional flavonoids (quercetin and kaempferol derivatives) were identified in cold-induced PC*_cpl1_* plants, which were labeled according to Tohge *et al* (2005) (Figure 5).

**Table 2:** Flavonoid phytochemicals identified by LC-MS analysis.

Peak	R*t*	λmax	ESI-MS	MS2^a^	(µg/gFW)				fold^b^
	(min)	(nm)	[M-H] + (*m/z*)		B3*_cpl1_* 23°C	B3*_cpl1_* 4°C	PC*_cpl1_* 23°C	PC*_cpl1_* 4°C	
A5	25.16	281, 524	975	**727**, 690, 535, 473, 287	2.74	3.71	27.88	119.90	43.8
A8	12.07	280, 523	1137	**1093**, 975, 889, 535	2.71	6.03	10.92	135.53	50.0
A9	25.48	313, 533	1181	**1163**, 1092, **933**, 535, 287	2.75	3.43	5.80	20.30	7.4
A10	14.12	283, 535	1257	**1095**, 933, 449	2.42	3.55	27.88	22.61	9.3
A11	16.82	285, 535	1343	1299, **1095**, 535	4.81	15.78	31.77	223.34	46.4
F1	21.54	264, 341	579	**433**, 287	11.86	136.28	10.35	101.10	8.5
F2	15.19	266, 337	595	**433**, 287	4.23	67.99	3.54	25.57	6.0
F3	7.9	266, 347	741	595, 433	6.69	80.0	7.22	67.14	10.0
F4	15.61	255, 346	595	**449**, 303	0	0	11.47	20.25	N/D
F5	10.78	254, 344	611	**571**, 449, 303	0	15.73	0	26.76	N/D
F6	5.93	255, 351	757	611, **449**, 303	13.01	27.74	12.53	28.17	2.1

Plant extract was obtained from plants treated for 3 weeks at 4°C.

a Numbers in bold letters indicate major ions detected in MS2.

b Calculated as (PC*_cpl1_* 4°C/B3*_cpl1_* 23°C). N/D; not determined.

In untreated B3*_cpl1_* plants, only a small amount of anthocyanin and flavonoid derivatives were identified. Instead, a peak, which eluted at around 20 min and corresponded to the sinapate derivative S2 reported in Tohge et al (2005), dominated the HPLC chromatogram (320 nm) (Figure 5, top). However, since we were not able to identify this peak, we did not include this peak in our analyses. Untreated PC*_cpl1_* plants harboring the three-component system showed slight elevation of anthocyanins and quercetin derivative F4 before cold treatment, but not other flavonoids. Cold treatment strongly induced production of anthocyanins in PC*_cpl1_* but not B3*_cpl1_* plants. Peak A11 containing 3 acyl moieties and 4 glycosides, was the most abundant anthocyanin identified, accounting for 62–63% of the total anthocyanins. Cold treatment also induced accumulation of various flavonoids that were detected in the HPLC chromatogram. Interestingly, kaempferol derivatives F1, F2 and F3 accumulated in cold-treated B3*_cpl1_*, however, their level in PC*_cpl1_* did not reach as high as that in B3*_cpl1_* even after the cold treatment. Instead, quercetin derivatives F4, F5, and F6 accumulated at higher level in PC*_cpl1_*. Overall, total extracted anthocyanins (A5, A8, A9, A10, A11) and flavonoids (F1–F6) increased ∼33 times and 7.5 times, respectively, in cold-treated PC*_cpl1_* relative to untreated B3*_cpl1_*.

## Discussion

Here we report an inducible gene expression system and its use in modifying phytochemicals in the model plant *Arabidopsis thaliana*. Inducible production of phytochemicals is a strategy commonly used in industrial culturing processes, however, the concept has not been widely adopted in genetic engineering of plant metabolism. In this study, we used cold induction, which activates production of a subset of flavonoids to regulate anthocyanin production. Compared to the anthocyanin profile obtained from previous constitutive overexpression [18], the amount of individual anthocyanins in cold treated PC*_cpl1_* was up to 5 fold higher (Table 2). This was similar to the level obtained when *pap1-D* plants were exposed to light stress [8]. These observations indicated that ectopic *PAP1* overexpression by itself did not fully activate all rate limiting steps of anthocyanin biosynthesis, and further activation of the phenylpropanoid pathway required additional environmental signals. In our case, cold treatment induced kaempferol biosynthesis both in B3*_cpl1_* and PC*_cpl1_* plants, albeit PC*_cpl1_* plants produced less kaempferol and more anthocyanins and quercetins upon cold treatment. Apparently, cold treatment in PC*_cpl1_* induced a sufficient metabolic flow to dihydrokaempferol, for which F3′H successfully competes with FLS. This contrasts with the case of isoflavone synthase overexpression, where isoflavone was overproduced only when a competing pathway was turned off [23]. It has been proposed that flavonoid pathway enzymes form a multi-enzyme supercomplex and channel metabolites between active sites [24]. Perhaps, sufficient amount of native F3′H, which were induced by PAP1, can associate with the proposed enzyme complex even in the presence of FLS. Downstream, in contrast, FLS successfully competed with DFR and produced quercetin. The resulting phytochemical profile showed higher level of anthocyanins, kaempferols, and quercetins. This contrasts with the case of *pap1-D*, in which anthocyanin accumulation was accompanied with substantial decrease of kaempferols [18].

In this system, it was necessary to have all three components and cold treatment to produce large amount of anthocyanins and flavonoids. Plants with only two components, such as PC transformants and PB*_cpl1_* transformants, did not produce anthocyanins more than control plants (Table 1 and data not shown). Indeed, although the *PAP1* expression levels of some cold-induced PC plants and uninduced PC*_cpl1_* plants were similar, only uninduced PC*_cpl1_* plants showed elevated anthocyanin accumulation. Currently we attribute these differences to the expression levels of *PAP1* in each cell. Perhaps, although cold-induced PC plants and uninduced PC*_cpl1_* plants showed similar level of total *PAP1* mRNA, distribution of *PAP1* transcripts are different between these plants. *PAP1* expression in uninduced PC*_cpl1_* plants likely is more restricted to specific tissues like trichomes, whereas lower but even expression occurs in cold-induced PC plants. Such difference could render above-threshold level *PAP1* expression in some uninduced PC*_cpl1_* tissues but not in cold-induced PC plants. An alternative possibility is that *cpl1-2* mutation de-represses anthocyanin biosynthetic genes. Since we did not succeed in increasing anthocyanin in B3*_cpl1_* or PB*_cpl1_*, it is unlikely that *cpl1* upregulates anthocyanin biosynthetic genes by directly. However, the possibility that *cpl1* increased responsiveness of anthocyanin biosynthetic genes to the activation by PAP1 cannot be excluded.

In conclusion, we have demonstrated the effectiveness of a three-component system, which consists of *RD29a-PAP1*, *RD29a-CBF3*, and the *cpl1* mutation. Expression of *PAP1* using an inducible three-component system can minimize severe vegetative growth inhibition caused by the constitutive expression of transgenes. Unlike several inducible systems, such as dexamethasone inducible, the three component system described here did not require any constitutive expression of the system components, and therefore, would be more resistant to gene silencing. Since the *cpl1* mutation can enhance expression of other inducible promoters in addition to osmotic stress pathway genes [25], the three-component system with *cpl1* is expected to be applicable for other inducible promoter-transcription factor combinations.

## Methods

### Construction of expression cassettes

Primer sequences used in this research are listed in Table S1. cDNA fragments encoding Arabidopsis *PAP1* and *CBF3* were amplified using primer pairs [680, 681] and [678,679], respectively. The entry plasmid pEnRD29A-LUC was prepared by inserting an *RD29A-LUC* expression cassette [26] into pEntr2B (Invitrogen, CA). pEnRD29A-PAP1 and pEnRD29A-CBF3 were prepared by replacing luciferase ORF (*LUC*) with *PAP1* and *CBF3* coding sequences, respectively. A plasmid vector pFAJ3163 containing the *BAR* gene was provided by Dr. Cammue [27], and pFAJGW was prepared by replacing the *35S-GUS* cassette with a gateway cassette. A plasmid vector pMDC99 containing *HPT* [28] was provided by Arabidopsis Biological Resource Center. In order to prepare plant transformation binary plasmids, pEnRD29A-PAP1 and pEnRD29A-CBF3 were recombined using LR clonase (Invitrogen, CA) with pFAJGW and pMDC99, respectively.

### Plant growth condition

For in vitro culture, surface-sterilized seeds were sown on media containing 1/4×MS salts, 0.5% sucrose and 0.8% agar. After stratification for 2–4 days, plates were incubated at 23°C for 7 days under 16 hr light/8 hr dark cycle. For cold treatment, plates were then moved to 4°C and incubated for additional 4 days.

For growth and induction of anthocyanin accumulation on soil, seeds were sown directly on Metromix 366 potting media. After 2–4 days' stratification, plants were grown at 23°C for 3 weeks under a 16 hour light/8 hour dark cycle. For cold treatment, plants were then moved to 4°C and grown for specified periods under the 16 hour light/8 hour dark cycle.

### Arabidopsis double transformation

Binary plasmids pMDC-CBF3 and pFAJ-PAP1 were transformed into *Agrobacterium tumefaciens* GV3101 and ABI, respectively. Empty vector controls (pBIB-HYG, pFAJ3163) were also transformed into Agrobacteria. In order to transform Arabidopsis, bacterial suspensions were prepared in solution containing 5% sucrose and 0.03% Silwet L-77. Mixtures (1∶1) of suspensions were prepared in following combinations: [pMDC-CBF3 and pFAJ-PAP1 (PC)], [pFAJ-PAP1 and pBIB (PB)], [pMDC-CBF3 and pFAJ3163 (C3)], or [pBIB and pFAJ3163 (B3)] and applied to flower buds of Arabidopsis wild type and *cpl1-2* mutants. Resulting T_1_ seeds of eight genotypes were harvested separately.

For selection of double transformants, first, hygromycin-resistant transformants were selected on media containing 1/4×MS salts, 0.5% sucrose, 30 µg/ml hygromycin B, 100 µg/ml cefotaxime and 0.8% agar. Sixty lines of each selected genotype of T_1_ plants were then transplanted to the soil and sprayed with 30 µg/ml phosphinothricine to identify PPT^R^ transformants. Thirty T_1_ double transformants of each combination were harvested and subjected to Hyg^R^ and PPT^R^ selection again to obtain single copy T_2_ transformants. T_3_ plants were tested again to identify transformants homozygous for both Hyg^R^ and PPT^R^. T_4_ plants that contained single copy T-DNA for both transgenes as homozygous state were used for further analysis.

### Gene Expression Analyses by RT-qPCR

Total RNA was isolated using Trizol reagent (Invitrogen, CA). RNA samples resuspended in 50 µl of water were treated with 7.5 unit of DNase I (Qiagen, MD) for 60 min at 37°C, and re-purified with RNeasy plant mini kit (Qiagen). Quantitative reverse-transcription PCR (RT-qPCR) was performed as described previously [29]. The absence of genomic DNA contamination was confirmed using minus-reverse-transcriptase controls. The data were processed as described previously. For screening of transgenic plants, specific primer pairs for RT-qPCR analyses were: [Y888, Y889] for RD29A, [Y882, Y883] for PAP1 and [Y884, Y885] for CBF3. For time-course analyses, primer pairs are as followings: [Y980, Y981] for RD29A, [Y976, Y977] for PAP1, [Y978, Y979] for CBF3, [Y958, Y959] for PAL1, [Y960, Y961] for CHS, [Y968, Y969] for CHI, [Y962, Y963] for DFR, [Y970, Y971] for F3′H, [Y1006, Y1007] for ANS, [Y996, Y997] for FLS1, [Y1000, Y1001] for FLS3, [Y1002, Y1003] for UGT73B2, and [Y1004, Y1005] for UGT78D2.

### Phytochemical identification and quantification by LC-MS

For spectrophotometric quantification of total anthocyanin content, one gram of leaf samples were processed as described [30]. The anthocyanin contents were calculated as cyanidin 3-glucoside equivalent.

For LC-MS analysis of anthocyanins and flavonoids, one gram of leaf samples were ground in liquid nitrogen, and extracted with five grams of methanol∶water∶acetic acid (9∶10∶1) at 4°C for 24 h in dark on a shaker at 120 rpm. Extracts were centrifuged at 10,000 g at 4°C for 20 min. The supernatant was filtered with a 0.22 µm nylon filter (Fisher Scientific, PA).

Individual compounds were identified based on retention time, UV spectra and their mass per charge ratio using LC-MS as described previously [18]. Compounds were quantified as equivalents of cyanidin-3-glucoside, kaempferol, or quercetin, depending on their core compounds. Standard curves were performed for each individual core compound. The same conditions were used for phytochemical identification and quantification.

Chromatographic separation was performed on a LCQ Deca XP Max LC-MS/MS system (Thermo Finnigan, CA) equipped with an autosampler, a Surveyor 2000 quaternary pump and a UV 2000 PDA detector, using an 150×2.00 mm Synergi 4 µ Hydro RP 80A column (Phenomenex, Torrance, CA) and a guard column of the same chemistry. Individual compounds were identified based on retention time, UV spectra and their mass per charge ratios using LC-MS as described previously [18]. Elution gradient was formed with solvent A [acetonitrile∶water∶formic acid (13∶87∶1)] and solvent B [acetonitrile∶formic acid (100∶1)]. Separations were achieved by a linear gradient with A and B: 0 min 100% A, 8 min 97% A, 13 min 95% A, 21 min 95% A, 23 min 73% A, 28 min 78% A, 30 min 100% B, 35 min 100% B. The flow rate was 200 µl/min. The injection volume was 10 µl.

Samples were delivered to the LCQ MS by electrospray ionization (ESI). Conditions for analysis in positive ion mode were: spray voltage at 5.0 KV, sheath gas flow rate at 50 arbitrary units, auxiliary gas flow rate at 3.0 arbitrary units, capillary temperature at 275°C, and capillary voltage at 10 V. Spectra were scanned over a mass range of m/z 180–2000 at 3 scans sec^−1^. Helium was used as collision gas and collision energy was set at 30%. MS^2^ and MS^3^ analyses were used during the identification.

## Supporting Information

Table S1 Sequences of primers.
(DOC)
