# Supplementary material for: A Three-Component Gene Expression System and Its Application for Inducible Flavonoid Overproduction in Transgenic Arabidopsis thaliana
Source: PLoS One. 2011 Mar 8;6(3):e17603. doi: 10.1371/journal.pone.0017603 (PMC3050924; doi:10.1371/journal.pone.0017603)
Supplement: Table S1 — Sequences of primers. (DOC) [file pone.0017603.s001.doc]

**Table S1.** Sequences of primers

| Name | Sequence |
| --- | --- |
| 680 | AAGGTCGACCATGGAGGGTTCGTCCA |
| 681 | TTTCCCGGGCTAATCAAATTTCACAG |
| 678 | AGGGTCGACCATGAACTCATTTTCTG |
| 679 | TTTCCCGGGTTAATAACTCCATAACG |
| Y888 | CGGAGCATGAGTCATTGAACACTCCG |
| Y889 | ATTCATCATCACCACTTGGAGCA |
| Y882 | CGGTTATCTGAGTAAGAAACATGAACCG |
| Y883 | TTTAGTGCCGGTGTTGTAGGAAT |
| Y884 | CGTGTATGGTGGAGGCTATTTACACG |
| Y885 | CCAACAAACTCGGCATCTCAAA |
| Y976 | TCTAGGGAATAGGTGGTCTTTA |
| Y977 | CACGGTTCATGTTTCTTACTC |
| Y978 | GAAATGTTTGGCTCCGATTAC |
| Y979 | TCACGAAACTTCTTACGACC |
| Y980 | CGGCTGTTTCAGACTATCTT |
| Y981 | ACTCCTCCAGTTTCTTCTTTG |
| Y958 | GTATGAACAAAGGCACTGATAG |
| Y959 | CGGCGTTAAGGAATCTAATAAG |
| Y960 | ACCTCAAGGAGAAGTTCAAG |
| Y961 | TTTCCTTGAGGAATTCCTCC |
| Y968 | ACTTACCATTTCTCAGCCAA |
| Y969 | TTCTCCACCTTCAGTTTCTC |
| Y962 | AAGATCCTGAGAACGAAGTG |
| Y963 | CTTTGCCTTAACACATGCTT |
| Y970 | CTTACCTTCAGGCGGTTATC |
| Y971 | GTTCGTCAATAGAGTCGATCC |
| Y1006 | CTTCTTTCATCTTGCGTATCC |
| Y1007 | CTCGTTGCTTCTATGTAATCAC |
| Y996 | CACTCTTCTTGTTCCTAACGA |
| Y997 | CCCATTACTCAACCTCAGAATC |
| Y1000 | CCTCCAGAATACATTGAAGTGA |
| Y1001 | ATGCCTTAAACCTAGTCCTTC |
| Y1002 | CAAATTCAGTCATTTATGTTTCCTTT |
| Y1003 | CATTCTTCTCTATCATCTTTGGTTT |
| Y1004 | GAGATAAATGCGTCGTGGATT |
| Y1005 | GCTCACCTACTTCTTTGACAC |
